
JOURNAL INFORMATION
==============================
NLM Title Abbreviation: ScientificWorldJournal
Journal ID: ScientificWorldJournal
NLM Journal ID: 101131163
Journal ID: TSWJ

The Scientific World Journal

ISSN: 2356-6140
EISSN: 1537-744X
Publisher: Wiley

ARTICLE INFORMATION
==============================
PMCID: PMC6084261
PMID: 30147617
DOI: 10.1100/tsw.2001.241
Article ID: 891649
Article version: 1
Subjects: Short Report

Execution of Necrotic-Like Cell Death in Caenorhabditis elegans Requires Cathepsin D Activity

Tavernarakis Nektarios *
Xu Keli
Driscoll Monica
Department of Molecular Biology and Biochemistry, Rutgers University, 604 Allison Road, Piscataway NJ 08855, USA
*Nektarios Tavernarakis: tavernarakis@mbcl.rutgers.edu
Publication date: 2001
Electronic publication date: 2001 Nov 10
Volume: 1
First page: 139
Last page: 139
Copyright: Copyright © 2001 Nektarios Tavernarakis et al.
Copyright year: 2001
License: This is an open access article distributed under the Creative Commons Attribution License, which permits unrestricted use, distribution, and reproduction in any medium, provided the original work is properly cited.
License URL: https://creativecommons.org/licenses/by/3.0/

==============================
Miami *Nature Biotechnology* Short Reports

*TheScientificWorld* (2001) 1 (S3), 139SR

ISSN 1532-2246; DOI 10.1100/tsw.2001.241

### EXECUTION OF NECROTIC-LIKE CELL DEATH IN *CAENORHABDITIS ELEGANS* REQUIRES CATHEPSIN D ACTIVITY

Nektarios Tavernarakis*, Keli Xu, and Monica Driscoll

Department of Molecular Biology and Biochemistry, Rutgers University, 604 Allison Road, Piscataway NJ 08855
* tavernarakis@mbcl.rutgers.edu

**INTRODUCTION.** Inappropriate cell death underlies the pathology of many human and animal diseases. In particular, premature neuronal cell death plays a significant role in several late onset degenerative disorders such as Alzheimer's Disease and Amyotrophic Lateral Sclerosis (1). The mechanisms of execution of pathological or necrotic cell death are not clear, but a detailed molecular model of inherited neurodegenerative conditions, identified in the nematode *Caenorhabditis elegans*, is emerging and may provide a means of identifying a conserved pathway for pathological cell death in humans (2). Gain-of-function mutations in several, specific, *C. elegans* ion channel genes encode hyperactive channels that induce necrotic-like death of the neurons that express these channel genes. For example, dominant mutations in the *mec-4* gene (*mec*hanosensory; *mec-4* allele *u231* or *d*: *d*ominant) induce degeneration of six touch receptor neurons required for the sensation of gentle touch to the body (3,4). We are investigating the requirements for the progression and execution of degenerative cell death inflicted by *mec-4(d)* and other insults in *C. elegans*. To highlight the molecular events that transpire during neurodegeneration we have embarked on an effort to define conditions that block or interfere with necrotic-like cell death. We report here that Cathepsin D, which is an aspartyl protease, is required for the execution of necrotic-like cell death in *C. elegans*.

**RESULTS.** Several mutant nematode strains have been found to exhibit up to 90% reduced Cathepsin D protease activity (5). We assessed the requirement for Cathepsin D activity in necrotic-like cell death induced by *mec-4(d)*, by testing the strains *cad-1* alleles *j2* and *j14*, *daf-4(e1364)*, *mec-8(e398)* and *unc-52(su250)* (known to have 80%-90% reduced Cathepsin D proteolytic activity) for effects in touch neuron degeneration. As shown in Table 1, necrotic-like cell death induced by *mec-4(d)* was significantly blocked in these genetic backgrounds. Starvation is another condition that leads to significant reduction of Cathepsin D activity in *C. elegans* (6). We have observed that starvation also interferes with necrotic-like cell death induced by *mec-4(d)* (Table1). To further demonstrate the involvement of Cathepsin D proteolytic activity in neurodegeneration, we assayed the effects of Pepstatin A, a potent and specific inhibitor of Cathepsin D on *mec-4(d)* induced necrotic-like cell death. As shown in Table 1, Pepstatin A significantly blocked neurodegeneration. There are several genes in the completely sequenced *C. elegans* genome that encode putative Cathepsin D proteases. The most highly similar to biochemically characterized mammalian aspartyl proteases has been designated *asp-4* (7). We employed dsRNA interference to quench *asp-4* expression and to determine the effects on neurodegeneration. Results presented in Table 1 indicate that ASP-4

activity is required for necrotic-like cell death induced by *mec-4(d)*. Finally we tested the ability of *asp-4* to suppress the effects of Cathepsin D deficient genetic backgrounds on *mec-4(d)* induced necrotic-like cell death, when overexpressed. As shown in Table 1, overexpression of *asp-4* in a *mec-4(d);cad-1(j2)* completely reversed the suppressing effects of *cad-1(j2)* on *mec-4(d)*induced neurodegeneration.

**Table 1.** Cathepsin D is required for execution of necrotic-like cell death in *C. elegans*.

| Strain | % vacuoles |
|---|---|
| *mec-4(d)* | 94±3 |
| *mec-4(d);cad-1(j2)* | 33±6 |
| *mec-4(d);cad-1(j14)* | 41±5 |
| *mec-4(d);daf-4(e1364)* | 36±6 |
| *mec-4(d);mec-8(e398)* | 49±7 |
| *mec-4(d);unc-52(su250)* | 43±4 |
| *mec-4(d)* Starvation | 68±9 |
| *mec-4(d)* Pepstatin A | 32±8 |
| *mec-4(d) asp-4*$_{\mathrm{dsRNAi}}$ | 42±9 |
| *mec-4(d);cad-1(j2) asp-4*$^{\mathrm{High}}$ | 89±8 |

**DISCUSSION.** Aspartyl proteases such as Cathepsin D are mostly lysosomal enzymes but they have also been found to function in the cytoplasm. Among several metabolic pathways, Cathepsin D has been implicated in endocytic degradation of proteins (8). Interestingly, degenerative cell death involves extensive plasma membrane internalization that resembles endocytosis (9). Such a process may require the function of Cathepsin D. Our results suggest that Cathepsin D activity plays a pivotal role in the disorganization and destruction of the cellular content during neurodegeneration that has been inflicted by *mec-4(d)* in *C. elegans*. To determine how general the requirement for aspartyl protease activity is, we are investigating the involvement of Cathepsin D in necrotic-like cell death, induced by insults other than *mec-4(d)*, in the nematode. If a central role for cathepsin D in the execution of degenerative cell death is established, such proteases could become attractive therapeutic targets that when inactivated might significantly delay or block the progression of inappropriate cell death.

**REFERENCES.**

1. Honig, L.S. and Rosenberg, R.N. (2000) Am. J. Med. 108, 317-330
2. Chalfie, M. and Wolinsky, E. (1990) Nature 345, 410-416
3. Driscoll, M. and Chalfie, M. (1991) Nature 349, 588-593
4. Tavernarakis, N. and Driscoll, M. (1997) Annu. Rev. Physiol. 59, 659-689
5. Jacobson, L.A., Jen-Jacobson, L., Hawdon, J.M., Owens, G.P., Bolanowski, M.A., Emmons, S.W., Shah, M.V., Pollock, R.A., and Conklin, D.S. (1988) Genetics 119, 355-363
6. Sarkis, G.J., Ashcom, J.D., Hawdon, J.M., and Jacobson, L.A. (1988) Mech. Ageing Dev. 45, 191-201
7. Tcherepanova, I., Bhattacharyya, L., Rubin, C.S., and Freedman, J.H. (2000) J. Biol. Chem. 275, 26359-26369

8. Blum, J.S., Fiani, M.L., and Stahl, P.D. (1991) J. Biol. Chem. 266, 22091-22095
9. Hall, D.H., Gu, G., Garcia-Anoveros, J., Gong, L., Chalfie, M., and Driscoll, M. (1997) J. Neurosci. 17, 1033-1045
